# Supplementary material for: Prevalence and distribution pattern of Cryptosporidium spp. among pre-weaned diarrheic calves in the Republic of Korea
Source: PLoS One. 2021 Nov 15;16(11):e0259824. doi: 10.1371/journal.pone.0259824 (PMC8592421; doi:10.1371/journal.pone.0259824)

PLOS ONE

Prevalence and distribution of Cryptosporidium spp. among diarrheic calves in the Republic of Korea

--Manuscript Draft--

**Manuscript Number:**	PONE-D-21-26307
**Article Type:**	Research Article
**Full Title:**	Prevalence and distribution of Cryptosporidium spp. among diarrheic calves in the Republic of Korea
**Short Title:**	Different subtypes of C. parvum in calves with diarrhea
**Corresponding Author:**	Kyoung-Seong Choi<br>Kyungpook National University<br>Sangju, KOREA, REPUBLIC OF
**Keywords:**	Cryptosporidium; pre-weaned calves; diarrhea; C. parvum; gp60 subtypes
**Abstract:**	Cryptosporidium spp. are protozoan parasites that cause diarrhea in humans and animals worldwide. Data on the prevalence of Cryptosporidium spp. and its subtypes among calves in the Republic of Korea (KOR) are sparse. Hence, our study aimed to investigate the prevalence and the association between the age of calf and the identified Cryptosporidium spp. and to determine the genotypes/subtypes of Cryptosporidium spp. in pre-weaned calves with diarrhea in the KOR. A total of 460 diarrheic fecal samples were collected from calves aged 1−60 days and screened for Cryptosporidium spp. by the 18S rRNA gene. Species identification was determined by the sequencing analysis of the 18S rRNA gene, and C. parvum -positive samples were subtyped by sequence analysis of the 60-kDa glycoprotein (gp60) gene. Sequencing analysis based on the 18S rRNA gene revealed the presence of three Cryptosporidium spp., namely, C. parvum (n = 72), C. ryanae (n = 12), and C. bovis (n = 2). Co-infection by these species was not observed. The infection rate was the highest in calves aged 11−20 days (26.1%, 95% CI 17.1−35.1), whereas the lowest rate was observed in calves aged 21−30 days (7.7%, 95% CI 0.0−16.1). The prevalence of C. parvum was detected exclusively in calves aged ≤20 days, and the highest infection rate of C. ryanae was seen in calves ≥31 days of age. The occurrence of C. parvum (χ^2 = 25.300, P = 0.000) and C. ryanae (χ^2 = 18.020, P = 0.001) was significantly associated with the age of the calves. Eleven different subtypes belonging to the family IIa were recognized: IIaA14G1R1, IIaA14G3R1, IIaA15G1R1, IIaA15G2R1, IIaA16G4R1, IIaA17G3R1, IIaA17G4R1, IIaA18G3R1, IIaA19G1R1, IIaA19G3R1, and IIaA19G4R1. Except for two (IIaA18G3R1 and IIaA15G2R1) subtypes, nine subtypes were first identified in calves with diarrhea in the KOR. IIaA18G3R1 was the most frequently detected subtype (72.2% of calves), followed by IIaA17G3R1 (5.6%), and IIaA15G2R1 (4.2%) and IIaA19G4R1 (4.2%). These results suggest that the prevalence of Cryptosporidium spp. is significantly associated with calf age. Furthermore, the findings demonstrate the high genetic diversity of C. parvum and the widespread occurrence of zoonotic C. parvum in pre-weaned calves. Hence, calves are a potential source of zoonotic transmission with considerable public health implications.
**Order of Authors:**	Dong-Hun Jang
	Hyung-Chul Cho
	Seung-Uk Shin
	Eun-Mi Kim
	Yu-Jin Park
	Sunwoo Hwang
	Jinho Park
	Kyoung-Seong Choi
**Additional Information:**	

Powered by Editorial Manager® and ProduXion Manager® from Aries Systems Corporation

Question	Response
**Financial Disclosure**<br><br>Enter a financial disclosure statement that describes the sources of funding for the work included in this submission. Review the submission guidelines for detailed requirements. View published research articles from *PLOS ONE* for specific examples.<br><br>This statement is required for submission and **will appear in the published article** if the submission is accepted. Please make sure it is accurate.<br><br>**Unfunded studies**<br>Enter: *The author(s) received no specific funding for this work.*<br><br>**Funded studies**<br>Enter a statement with the following details:<br>• Initials of the authors who received each award<br>• Grant numbers awarded to each author<br>• The full name of each funder<br>• URL of each funder website<br>• Did the sponsors or funders play any role in the study design, data collection and analysis, decision to publish, or preparation of the manuscript?<br>• **NO** - Include this sentence at the end of your statement: *The funders had no role in study design, data collection and analysis, decision to publish, or preparation of the manuscript.*<br>• **YES** - Specify the role(s) played.<br><br>* typeset	Kyoungg-Seong CHoi<br>: This research was supported by the Korea Institute of Planning and Evaluation for Technology in Food, Agriculture, and Forestry (IPET) (Grant No. 321016-01-1-HD020). The funders had no role in study design, data collection and analysis, decision to publish, or preparation of the manuscript.
**Competing Interests**<br><br>Use the instructions below to enter a competing interest statement for this submission. On behalf of all authors, disclose any competing interests that could be perceived to bias this work—acknowledging all financial support and any other relevant financial or non-financial competing interests.	No: The authors have declared that they no competing interests exit.

Powered by Editorial Manager® and ProduXion Manager® from Aries Systems Corporation

This statement is required for submission and **will appear in the published article** if the submission is accepted. Please make sure it is accurate and that any funding sources listed in your Funding Information later in the submission form are also declared in your Financial Disclosure statement.<br><br>View published research articles from *PLOS ONE* for specific examples.<br><br>**NO authors have competing interests**<br><br>Enter: *The authors have declared that no competing interests exist.*<br><br>**Authors with competing interests**<br><br>Enter competing interest details beginning with this statement:<br><br>*I have read the journal's policy and the authors of this manuscript have the following competing interests: [insert competing interests here]*<br><br>* typeset	
**Ethics Statement**<br><br>Enter an ethics statement for this submission. This statement is required if the study involved:<br><br>• Human participants<br>• Human specimens or tissue<br>• Vertebrate animals or cephalopods<br>• Vertebrate embryos or tissues<br>• Field research<br><br>Write "N/A" if the submission does not require an ethics statement.<br><br>General guidance is provided below. Consult the submission guidelines for detailed instructions. **Make sure that all information entered here is included in the Methods section of the manuscript.**	All animal procedures were conducted according to ethical guidelines for the use of animal samples, and were approved by the Jeonbuk National University (Institutional Animal Care and Use Committee Decision No. CBNU 2020-052). All procedures and possible consequences were explained to the managers of the surveyed farm, and written consent was obtained.

Powered by Editorial Manager® and ProduXion Manager® from Aries Systems Corporation

**Format for specific study types**<br><br>**Human Subject Research (involving human participants and/or tissue)**<br>• Give the name of the institutional review board or ethics committee that approved the study<br>• Include the approval number and/or a statement indicating approval of this research<br>• Indicate the form of consent obtained (written/oral) or the reason that consent was not obtained (e.g. the data were analyzed anonymously)<br><br>**Animal Research (involving vertebrate animals, embryos or tissues)**<br>• Provide the name of the Institutional Animal Care and Use Committee (IACUC) or other relevant ethics board that reviewed the study protocol, and indicate whether they approved this research or granted a formal waiver of ethical approval<br>• Include an approval number if one was obtained<br>• If the study involved *non-human primates*, add *additional details* about animal welfare and steps taken to ameliorate suffering<br>• If anesthesia, euthanasia, or any kind of animal sacrifice is part of the study, include briefly which substances and/or methods were applied<br><br>**Field Research**<br><br>Include the following details if this study involves the collection of plant, animal, or other materials from a natural setting:<br>• Field permit number<br>• Name of the institution or relevant body that granted permission	
**Data Availability**<br><br>Authors are required to make all data underlying the findings described fully available, without restriction, and from the time of publication. PLOS allows rare exceptions to address legal and ethical concerns. See the PLOS Data Policy and FAQ for detailed information.	Yes - all data are fully available without restriction

Powered by Editorial Manager® and ProduXion Manager® from Aries Systems Corporation

A Data Availability Statement describing where the data can be found is required at submission. Your answers to this question constitute the Data Availability Statement and **will be published in the article**, if accepted.<br><br>**Important:** Stating 'data available on request from the author' is not sufficient. If your data are only available upon request, select 'No' for the first question and explain your exceptional situation in the text box.<br><br>Do the authors confirm that all data underlying the findings described in their manuscript are fully available without restriction?	
**Describe where the data may be found in full sentences. If you are copying our sample text, replace any instances of XXX with the appropriate details.**<br><br>• If the data are **held or will be held in a public repository**, include URLs, accession numbers or DOIs. If this information will only be available after acceptance, indicate this by ticking the box below. For example: *All XXX files are available from the XXX database (accession number(s) XXX, XXX.*).<br>• If the data are all contained **within the manuscript and/or Supporting Information files**, enter the following: *All relevant data are within the manuscript and its Supporting Information files.*<br>• If neither of these applies but you are able to provide **details of access elsewhere**, with or without limitations, please do so. For example:<br><br>*Data cannot be shared publicly because of [XXX]. Data are available from the XXX Institutional Data Access / Ethics Committee (contact via XXX) for researchers who meet the criteria for access to confidential data.*<br><br>*The data underlying the results presented in the study are available*	All relevant data are within the manuscript and its Supporting Information files.

Powered by Editorial Manager® and ProduXion Manager® from Aries Systems Corporation

from (include the name of the third party and contact information or URL).<br>• This text is appropriate if the data are owned by a third party and authors do not have permission to share the data.<br><br>* typeset	
Additional data availability information:	Tick here if the URLs/accession numbers/DOIs will be available only after acceptance of the manuscript for publication so that we can ensure their inclusion before publication.

Powered by Editorial Manager® and ProduXion Manager® from Aries Systems Corporation

Manuscript Click here to access/download;Manuscript;PLos One.docx

Prevalence and distribution of *Cryptosporidium* spp. among diarrheic calves in the Republic of Korea

Dong-Hun Jang[1], Hyung-Chul Cho[1], Seung-Uk Shin[1], Eun-Mi Kim[1], Yu-Jin Park[2], Sunwoo Hwang[1], and Jinho Park[3], Kyoung-Seong Choi[1,2*]

[1]Department of Animal Science and Biotechnology, College of Ecology and Environmental Science, Kyungpook National University, Sangju 37224, Republic of Korea

[2]Department of Horse/Companion and Wild animals, College of Ecology and Environmental Science, Kyungpook National University, Sangju 37224, Republic of Korea

[3]College of Veterinary Medicine, Jeonbuk National University, Iksan 54596, Republic of Korea

*Correspondence should be addressed to:

Kyoung-Seong Choi, DVM, MS, PhD

College of Ecology and Environmental Science

Kyungpook National University

Sangju 37224, Republic of Korea

Tel: 82-54-530-1222

Fax: 82-54-530-1959

E-mail: kschoi3@knu.ac.kr

Abstract

Cryptosporidium spp. are protozoan parasites that cause diarrhea in humans and animals worldwide. Data on the prevalence of *Cryptosporidium* spp. and its subtypes among calves in the Republic of Korea (KOR) are sparse. Hence, our study aimed to investigate the prevalence and the association between the age of calf and the identified *Cryptosporidium* spp. and to determine the genotypes/subtypes of *Cryptosporidium* spp. in pre-weaned calves with diarrhea in the KOR. A total of 460 diarrheic fecal samples were collected from calves aged 1–60 days and screened for *Cryptosporidium* spp. by the 18S rRNA gene. Species identification was determined by the sequencing analysis of the 18S rRNA gene, and *C. parvum*-positive samples were subtyped by sequence analysis of the 60-kDa glycoprotein (*gp60*) gene. Sequencing analysis based on the 18S rRNA gene revealed the presence of three *Cryptosporidium* spp., namely, *C. parvum* (*n* = 72), *C. ryanae* (*n* = 12), and *C. bovis* (*n* = 2). Co-infection by these species was not observed. The infection rate was the highest in calves aged 11–20 days (26.1%, 95% CI 17.1–35.1), whereas the lowest rate was observed in calves aged 21–30 days (7.7%, 95% CI 0.0–16.1). The prevalence of *C. parvum* was detected exclusively in calves aged ≤20 days, and the highest infection rate of *C. ryanae* was seen in calves ≥31 days of age. The occurrence of *C. parvum* (χ^2 = 25.300, *P* = 0.000) and *C. ryanae* (χ^2 = 18.020, *P* = 0.001) was significantly associated with the age of the calves. Eleven different subtypes belonging to the family IIa were recognized: IIaA14G1R1, IIaA14G3R1, IIaA15G1R1, IIaA15G2R1, IIaA16G4R1, IIaA17G3R1, IIaA17G4R1, IIaA18G3R1, IIaA19G1R1, IIaA19G3R1, and IIaA19G4R1. Except for two (IIaA18G3R1 and IIaA15G2R1) subtypes, nine subtypes were first identified in calves with diarrhea in the KOR. IIaA18G3R1 was the most frequently detected subtype (72.2% of calves), followed by IIaA17G3R1 (5.6%),

and IIaA15G2R1 (4.2%) and IIaA19G4R1 (4.2%). These results suggest that the prevalence of *Cryptosporidium* spp. is significantly associated with calf age. Furthermore, the findings demonstrate the high genetic diversity of *C. parvum* and the widespread occurrence of zoonotic *C. parvum* in pre-weaned calves. Hence, calves are a potential source of zoonotic transmission with considerable public health implications.

Keywords: *Cryptosporidium*, pre-weaned calves, diarrhea, *C. parvum*, *gp60* subtypes

Introduction

Cryptosporidium spp. are protozoan parasites that cause mild-to-severe diarrhea in humans and a wide range of animals [1]. Infections with these parasites occur via the fecal-oral route either by direct contact with infected animals or by the ingestion of infective oocysts from contaminated water or food [2-5]. To date, 40 *Cryptosporidium* spp. have been described [6], and among them, four species, namely, *C. andersoni*, *C. bovis*, *C. parvum*, and *C. ryanae*, have been identified in cattle. The distribution of these species is known to vary according to age [4, 7]. In particular, *C. parvum* is one of the most important pathogens causing diarrhea in neonatal calves worldwide and leads to severe economic losses owing to poor growth, decreased productivity, and even death [8]. Moreover, *C. parvum* is the major pathogenic species that affects humans [9, 10]. Unlike *C. parvum*, *C. bovis* and *C. ryanae* usually infect post-weaned calves and yearlings without causing illness, and *C. andersoni* is mainly found in adult cattle [11-13]. The pathogenicity of *C. bovis*, and *C. ryanae* in post-weaned calves has not been established [9]. The oocysts of *C. parvum*, *C. bovis*, and *C. ryanae* are similar in size and shape. While *C. ryanae* is smaller than the others and requires molecular methods for its determination [14, 15], *C. andersoni* is larger in size and infects the abomasum [16].

According to the subtyping of *C. parvum* based on sequence analysis of the 60-kDa glycoprotein (*gp*60) gene, Ⅱa and Ⅱd subtypes have been detected in both humans and calves and can cause zoonotic cryptosporidiosis [17]. The Ⅱa subtype is mostly identified in calves, and IIaA15G2R1 is the predominant subtype [7] globally, including the Republic of Korea (KOR) [18]. The IId subtype is usually found in lambs and goat kids [4, 19] and has been described in calves in some countries such as Sweden, Turkey, Egypt, and China [20-23]. To date, most investigations of cryptosporidiosis in calves caused by *C. parvum* have focused on

the IIa subtype in most countries. However, there are a few studies on *C. parvum* subtypes in calves in the KOR [18, 24].

Cryptosporidium parvum infects the intestinal mucosa and accounts for over 90% of *Cryptosporidium* infections in neonatal calves [23]. In contrast, in pre-weaned calves, the prevalence of *C. bovis* and *C. ryanae* and their effects on causing diarrhea remain unclear. Several studies have reported that *C. bovis* and *C. ryanae* are present in pre-weaned calves [23, 25, 26] and that *C. ryanae* infections are particularly associated with moderate diarrhea in pre-weaned calves [23]. However, little is known about the association between *C. bovis* and diarrhea. In addition, a previous study has indicated that the high prevalence of *C. bovis* and *C. ryanae* in hemorrhagic diarrhea was found in the KOR [24]. Nevertheless, the pathogenicity of these organisms is still unclear. Therefore, this study aimed to investigate the prevalence of *Cryptosporidium* spp. in pre-weaned calves with diarrhea and to evaluate the association between the age of calf and the identified *Cryptosporidium* spp. Furthermore, we intended to determine genotype of *Cryptosporidium* spp. and subtyping of *C. parvum* in calves in the KOR and to assess the significance of calves as a source of human infections.

Materials and methods

Ethics statement

All animal procedures were conducted according to ethical guidelines for the use of animal samples, and were approved by the Jeonbuk National University (Institutional Animal Care and Use Committee Decision No. CBNU 2020-052). All procedures and possible consequences were explained to the managers of the surveyed farm, and written consent was obtained.

Sample collection

Between August 2019 and August 2020, fresh fecal samples were collected directly from the rectum of 460 diarrheic pre-weaned calves (up to 60 days of age) by an experienced veterinarian using sterile plastic gloves in 11 different farms located in the KOR. The samples were placed in labeled sterile plastic tubes and transported to the Animal Immunology Laboratory of Kyungpook National University in a cooler with ice packs. Upon arrival, sampling date, age, animal identification number, and fecal consistency (pasty, loose, watery, or hemorrhagic) were recorded for each animal. The collected feces were mostly pasty or loose. Prior to DNA extraction, all feces were stored at 4°C for no more than 2 days. The fecal samples were divided according to age as follows; 1–10 days (n = 271), 11–20 days (n = 92), 21–30 days (n = 39), and ≥31 days (n = 58). No microscopic examination was performed.

DNA extraction, molecular analysis, and sequencing

DNA was extracted from 2[illegible] mg of each fecal sample using the QIAamp Fast DNA Stool Mini Kit (Qiagen, Hilden, Germany) according to the manufacturer's instructions and

frozen at −20℃ until use. The *Cryptosporidium* spp. were first tested using the 18S rRNA gene [27]. Samples that yielded positive results for *Cryptosporidium* spp. were further tested using species-specific primers [24]. After that, *C. parvum* was again subtyped using a nested PCR targeting the 60-kDa glycoprotein (*gp60*) gene [4, 28]. All positive PCR products were purified using the AccuPower PCR Purification Kit (Bioneer, Daejeon, KOR) and employed for direct sequencing (Macrogen, Daejeon, KOR). The nucleotide sequences obtained in this study were analyzed using BioEdit (version 7.2.5) and compared with the reference sequences using the Basic Local Alignment Search Tool available at the National Center for Biotechnology Information database. To determine the subtype of *C. parvum*, nucleotide sequences were aligned using ClustalX and then analyzed via direct comparison with reference sequences from GenBank. Since the base sequences of *C. bovis* and *C. ryanae* are similar, all positive samples of the18S rRNA gene were separated by comparing the sequences. In this study, only samples showing a good sequencing result were considered positive for each *Cryptosporidium* spp. All nucleotide sequences generated in this study were deposited in the GenBank database with appropriate accession numbers (18S rRNA: MZ736386–MZ736399; *gp60*: MZ736314–MZ736385).

Statistical analysis

Statistical analysis was performed using SPSS Statistics 26 software package for Windows (SPSS Inc, Chicago, IL, USA). Chi-square test was used to determine the association between the prevalence of each species and age. A *p*-value of less than 0.05 was considered statistically significant.

Results

Prevalence of *Cryptosporidium* spp.

Among the 460 diarrheic fecal samples examined, 86 (18.7%) were positive for *Cryptosporidium* spp. on PCR analysis and sequencing based on the 18S rRNA gene. Three *Cryptosporidium* spp. were identified in pre-weaned Korean native calves (Table 1). Of these, *C. parvum* (15.7%, 72/460) was the most detected, followed by *C. ryanae* (2.6%, 12/460) and *C. bovis* (0.4%, 2/460). Co-infection of these species was not observed. The prevalence of the three *Cryptosporidium* spp. was compared according to the age groups. As shown in Table 1, the infection rate of *Cryptosporidium* spp. was highest in calves aged 11–20 days (26.1%, 95% CI 17.1–35.1), whereas the lowest infection rate was observed in calves aged 21–30 days (7.7%, 95% CI 0.0–16.1). All three *Cryptosporidium* spp. were detected only in calves aged 1–10 days (Table 1). The association between *Cryptosporidium* spp. and age-distribution was investigated. Interestingly, the identified *Cryptosporidium* spp. varied according to the age of the calves. *C. parvum* infection was detected exclusively in calves ≤20 days of age (Table 2). The prevalence peaked at the age of 11–20 days and decreased rapidly thereafter (Table 2). *C. parvum* infection was significantly associated with the age of the calves (χ^2 = 25.300, P = 0.000). Unlike *C. parvum*, *C. ryanae* was found in all age groups, and the highest infection rate was observed at ≥31 days of age (Table 2). *C. ryanae* infection also had a significant age-related distribution (χ^2 = 18.020, P = 0.001). In contrast, *C. bovis* was detected only in two calves aged 10 days and 35 days, and there was no statistical significance in the age-related distribution (P = 0.590).

Distribution of *Cryptosporidium* spp. and *C. parvum* subtypes

All 72 *C. parvum*-positive samples were successfully amplified and subtyped by

sequence analysis of the *gp60* gene. A total of 11 different subtypes belonging to the family IIa were identified (Table 3). Subtype family IId was not detected. The distinction of each subtype within the IIa was in the number of trinucleotide region of TCA and TGA repeats (i.e., had one copy of sequence ACATCA immediately after the trinucleotide repeats). As shown in Table 3, in pre-weaned Korean native calves, the most frequently detected subtype was IIaA18G3R1 (72.2%), followed by IIaA17G3R1 (5.6%), and then IIaA15G2R1 (4.2%) and IIaA19G4R1 (4.2%). Other subtypes, namely, IIaA14G1R1 (1.4%), IIaA14G3R1 (1.4%), IIaA15G1R1 (1.4%), IIaA16G4R1 (2.8%), IIaA17G4R1 (2.8%), IIaA19G1R1 (1.4%), and IIaA19G3R1 (2.8%) were also identified. There was not much of a correlation between calf age and a specific subtype. IIaA19G4R1 was observed only in calves aged 1−10 days, whereas IIaA17G3R1 was found exclusively in calves aged 11−20 days. There were more various subtypes found in calves aged 1−10 days (Table 3). The most predominant subtype, IIaA18G3R1, was seen in all ages. This is the first report on the various zoonotic subtypes circulating in the KOR.

Based on the 18S rRNA gene, 14 (12 *C. ryanae* and 2 *C. bovis*) sequences were obtained and compared with the published literature. Twelve sequences of *C. ryanae* showed 95.1%–100% similarity with each other. The *C. ryanae* sequences shared 95.7%–100% identity with those found in Austria, China, India, Thailand, and Japan. Two sequences of *C. bovis* shared 94.1% similarity. These sequences demonstrated 95.5%–96.2% identity with those identified previously in the KOR and had 91.9%–96.2% homology with those from Austria, USA, Japan, and China. Interestingly, differences in nucleotides between *C. ryanae* and *C. bovis* were observed. As shown in Fig. 1, the nucleotides in the six positions, i.e., 440, 460, 464–466, and 470 were different between the two species.

Discussion

Cryptosporidium, along with rotavirus, has been well recognized as the main pathogen causing diarrhea in neonatal calves worldwide [29]. Our findings established the prevalence of *Cryptosporidium* spp. in pre-weaned diarrheic calves according to age, and the presence of various zoonotic subtypes of *C. parvum* in the KOR were identified. In the present study, the overall prevalence of *Cryptosporidium* spp. was found to be 18.7%, which is higher than that reported previously in the KOR [18, 24, 30]. These variations could be explained by the age of the animals, time of sample collection, and the differences in geographical location. However, the percentage of *Cryptosporidium* spp.-positive samples found in our study was lower than that reported in other countries such as Germany (88.9%), Japan (83.8%), China (38.4%), Italy (38.8%), Colombia (26.6%), Argentina (22.5%), and Estonia (22.6%) [25, 31-36].

In this study, the presence of three *Cryptosporidium* spp. in pre-weaned Korean native calves was ascertained: *C. bovis*, *C. parvum*, and *C. ryanae*. Of them, *C. parvum* was the most predominant species in the KOR. This finding agrees with the results observed in several other countries [7, 25, 32, 35, 37, 38]. Most studies have proven that *C. parvum* mainly infects calves up to 1 month of age [32, 39-42]. The results of the present study demonstrated that *C. parvum* was detected only in calves aged ≤20 days, and the infection rate was the highest in calves aged 11–20 days. This observation is consistent with a previous study performed by our group [18]. According to our findings, *C. parvum* was not detected in calves over 21 days of age. It is speculated that calves in this age group are less susceptible to *C. parvum* infection owing to the partial development of the immune system with increasing age, which reduces the effects compared to the neonatal calves. Moreover, *C. parvum* is known to cause watery diarrhea [23, 29]. In this study, the number of animals with watery feces was small; hence, the association

with diarrhea was not evaluated. Although we were not able to compare the occurrence of *C. parvum* with the diarrhea status, *C. parvum* was found to be the causative agent of diarrhea in young calves. Our results suggest that *C. parvum* infection is attributed to the significant age-related distribution ($P = 0.000$). Consequently, *C. parvum* was strongly associated with diarrhea in calves aged ≤20 days.

Cryptosporidium ryanae was the second most frequently detected species in pre-weaned Korean native calves. In general, *C. ryanae* is often found in post-weaned calves [15]. The results revealed that *C. ryanae* was detected in all age groups with a low prevalence and that its occurrence increased with age. Especially, the infection rate of *C. ryanae* was the highest in calves aged ≥31 days. The prevalence of *C. ryanae* found in this study was similar to that of a previous study performed in the KOR [24]. Our observation confirmed that *C. ryanae* has an age-associated distribution, similar to *C. parvum*. A recent study has reported that *C. ryanae* was common in pre-weaned as well as post-weaned calves and that the infection was associated with the occurrence of moderate diarrhea in pre-weaned calves [23]. In contrast, other studies have shown that *C. ryanae* was not associated with diarrhea [26, 38, 43]. So far, the pathogenicity of *C. ryanae* is controversial. A previous study conducted in the KOR demonstrated that although it is not a single infection, the prevalence of *C. ryanae* was significantly high in hemorrhagic diarrhea [24]. We could not arrive at a conclusion regarding the correlation with diarrhea since the number of *C. ryanae*-positive samples from diarrheic calves was small. Hence, *C. ryanae* infection may cause diarrhea in calves ≥21 days of age and should be considered as a causative agent of diarrhea in this age group. Further studies are necessary to clarify the pathogenicity of *C. ryanae* in pre-weaned calves.

We found that the prevalence of *C. bovis* was the lowest in pre-weaned Korean native

calves. This observation is contradictory to the results reported by several studies in which *C. bovis* was the dominant species in pre-weaned calves [20, 44-47]. In this study, *C. bovis* was detected only in two calves aged 10 and 35 days. Several studies have stated that *C. bovis* is common in 2–3-week-old calves [41, 43]. However, our result signified that *C. bovis* was not detected in this age (Table 1). Cai et al. mentioned that *C. bovis* usually appears after weaning and that the infection can last weeks or months and contribute to the small increase in *Cryptosporidium* infection rates soon after weaning [26]. This observation may also explain the low prevalence of *C. bovis* in the present study. To date, information on the prevalence and clinical signs of *C. bovis* infection in both pre-weaned and post-weaned calves is very limited in the KOR. *C. bovis* could have probably been considered to be less important than *C. parvum* and therefore overlooked as an etiological agent of diarrhea in calves. Moreover, the results revealed that infection by *C. bovis*, unlike the two other species, was not age-related. Most importantly, the involvement of *C. bovis* in diarrhea remains unclear. Unlike *C. ryanae*, many studies have suggested that *C. bovis* was associated with diarrhea [23, 26, 38, 48]. However, infection by *C. bovis*/*C. ryanae* may lead to clinical signs owing to the presence of *C. parvum* [32]. Therefore, the prevalence and pathogenicity of *C. bovis* in pre-weaned and post-weaned calves must be investigated through large-scale epidemiological surveys.

C. parvum IIa family is common in humans as well as calves and is considered potentially zoonotic. To date, three *C. parvum* subtypes have been detected in calves in the KOR [18, 24], whereas one subtype (IIaA16G3R1) was not found in this study. In addition to the two subtypes (IIaA15G2R1 and IIaA18G3R1) described above, nine other subtypes (IIaA14G1R1, IIaA14G3R1, IIaA15G1R1, IIaA16G4R1, IIaA17G3R1, IIaA17G4R1, IIaA19G1R1, IIaA19G3R1, and IIaA19G4R1) that have not previously been detected in the

KOR were identified for the first time, showing the presence of high genetic diversity. Among them, IIaA18G3R1 was most commonly found in pre-weaned Korean native calves with diarrhea. This result is inconsistent with that of a previous study in which IIaA15G2R1 was shown as the predominant subtype [18]. This difference could be attributed to the fact that in the previous study, both normal and diarrheic feces were used and that IIaA15G2R1 was detected regardless of diarrhea [18]. Other variations are due to the differences in the season of sampling, regions, the number of samples, and herd management. IIaA15G2R1 has been known as the most prevalent *C. parvum* subtype infecting humans and cattle in many countries [7, 33, 49-53] and has also been detected in calves without diarrhea [18, 32, 54]. There seems to be no relationship between the subtype and diarrhea. In the present study, IIaA15G2R1 was detected only in three calves with diarrhea and the third frequent subtype along with IIaA19G4R1.

Here, IIaA18G3R1 was the dominant subtype that accounted for 72.2% of *C. parvum*-infected pre-weaned Korean native calves and was the frequent cause of human cryptosporidiosis, besides being reported in calves and foals [55-60]. The second common subtype in the KOR, IIaA17G3R1, has been found in calves and humans in several countries [61-65]. IIaA19G4R1 was the third frequent subtype identified in the pre-weaned Korean native calves and was also detected in small ruminants and fish as well as humans and calves [55, 64, 66-68]. Interestingly, all sequences belonging to the IIaA19G4R1 subtype were identical to those reported from other countries previously. These subtypes are considered to be the most common ones in calves in the KOR.

The other seven subtypes were also identified in pre-weaned Korean native calves with diarrhea, but their prevalence was relatively low. Subtypes IIaA14G1R1, IIaA14G3R1, and

IIaA15G1R1 were each detected in one calf. IIaA14G1R1 was identified in calves, goat kids, and humans [7, 12, 17, 19, 25, 33, 51, 52]. IIaA14G3R1 was found in humans, calf, lambs, and fresh molluscan shellfish [19, 25, 69, 70]. IIaA15G1R1 has been reported in humans [28, 51, 52, 71, 72] as well as in cattle and goat kids [22, 73-75]. Subtypes IIaA16G4R1 and IIaA17G4R1 were each found in two calves in the current study. Unlike the other subtypes, IIaA16G4R1 has so far been noted only in neonatal calf with diarrhea [76], which is consistent with our findings. Subtype IIaA16G4R1 has not yet been detected in humans; however, it cannot be excluded the possibility that this may represent a significant health risk. IIaA17G4R1 subtype has been identified in humans, cattle, and goats [31, 33, 59, 70, 76, 77] and has further been detected in diarrheic calves [31]. Finally, subtypes IIaA19G1R1 and IIaA19G3R1 have each been identified in one calf. IIaA19G1R1 has been reported in humans, cattle, and sheep [35, 52, 63, 78-80]. IIaA19G3R1 has been identified in humans, cattle, and deer [60, 81-84]. This study is the first to report the presence of various subtypes in pre-weaned calves in the KOR.

To detect *C. bovis* and *C. ryanae*, 18S rRNA and heat-shock protein 70 genes are generally used [15]. According to sequence analysis of the 18S rRNA gene, *C. bovis* and *C. ryanae* showed ≥99% identity, and it is not always possible to differentiate between them by PCR [85, 86]. However, in this study, we used only the 18S rRNA gene. Even without phylogenetic analysis, the difference between the two species could be confirmed by sequencing analysis. At the six nucleotide positions of 440, 460, 464–466, and 470, *C. bovis* had C, T, A, T, C, and A, while *C. ryanae* had T, C, G, C, T, and G. These positions are representative markers that distinguish *C. ryanae* from *C. bovis*. Our results suggest that these two species can be discerned using the 18S rRNA gene.

Conclusion

Our results confirm the presence of three *Cryptosporidium* spp. in pre-weaned calves with diarrhea: *C. bovis*, *C. parvum*, and *C. ryanae*. *C. parvum* was found to be the dominant species in young calves in the KOR. The occurrence of *C. ryanae* and *C. parvum*, but not *C. bovis*, in pre-weaned Korean native calves was significantly related to age; the prevalence of *C. parvum* decreased with age, whereas that of *C. ryanae* increased with age. The most frequently detected subtype in calves with diarrhea was IIaA18G3R1, which was responsible for zoonotic transmission. This is the first report to identify nine potentially zoonotic subtypes belonging to the family IIa, which have not previously been reported in cattle in the KOR. This study establishes the high genetic diversity of *C. parvum* in diarrheic calves and the widespread distribution of zoonotic *C. parvum* in the KOR. Therefore, the results emphasize that young calves may be a potential source of infection and may serve as an important zoonotic reservoir for human cryptosporidiosis.

Acknowledgements

Not applicable

Author Contributions

Conceptualization: Kyoung-Seong Choi, Jinho Park

Data curation: Kyoung-Seong Choi, Jinho Park

Formal analysis: Dong-Hun Jang, Hyung-Chul Cho, Seung-Uk Shin, Eun-Mi Kim[1], Yu-Jin Park, Sunwoo Hwang,

Methodology: Dong-Hun Jang, Hyung-Chul Cho, Seung-Uk Shin, Eun-Mi Kim, Yu-Jin Park, Sunwoo Hwang

Funding acquisition: Kyoung-Seong Choi

Writing – original draft: Kyoung-Seong Choi,

Data Availability Statement

All data generated or analyzed during this study are included in the article. The nucleotide sequence obtained in the present study has been deposited in the GenBank database under the accession numbers MZ736314–MZ736399.

Funding

This research was supported by the Korea Institute of Planning and Evaluation for Technology in Food, Agriculture, and Forestry (IPET) (Grant No. 321016-01-1-HD020). The funders had no role in study design, data collection and analysis, decision to publish, or preparation of the manuscript.

Competing interests

The authors have declared that they no competing interests exit.

References

1. Fayer R (2004) *Cryptosporidium*: a water-borne zoonotic parasite. Vet Parasitol 126: 37-56. https://doi.org/10.1016/j.vetpar.2004.09.004 PMID: 15567578
2. Xiao L, Ryan UM (2004) Cryptosporidiosis: an update in molecular epidemiology. Curr Opin Infect Dis 17:483-490. https://doi.org/10.1097/00001432-200410000-00014 PMID: 15353969
3. McLauchlin J, Amar C, Pedraza-Diaz S, Nichols GL (2000) Molecular epidemiological analysis of *Cryptosporidium* spp. in the United Kingdom: results of genotyping *Cryptosporidium* spp. in 1,705 fecal samples from humans and 105 fecal samples from livestock animals. J Clin Microbiol 38:3984-3990. https://doi.org/10.1128/JCM.38.11.3984-3990.2000 PMID: 11060056
4. Xiao L (2010) Molecular epidemiology of cryptosporidiosis: an update. Exp Parasitol 124:80-89. https://doi.org/10.1016/j.exppara.2009.03.018 PMID: 19358845
5. Wells B, Shaw H, Hotchkiss E, Gilray J, Ayton R, Green J, et al. (2015) Prevalence, species identification and genotyping *Cryptosporidium* from livestock and deer in a catchment in the Cairngorms with a history of a contaminated public water supply. Parasit Vectors 8:66. https://doi.org/10.1186/s13071-015-0684-x PMID: 25650114
6. Feng Y, Ryan UM, Xiao L (2018) Genetic diversity and population structure of *Cryptosporidium*. Trends Parasitol 34:997-1011. https://doi.org/10.1016/j.pt.2018.07.009 PMID: 30108020
7. Lichtmannsperger K, Harl J, Freudenthaler K, Hinney B, Wittek T, Joachim A (2020) *Cryptosporidium parvum*, *Cryptosporidium ryanae*, and *Cryptosporidium bovis* in samples from calves in Austria. Parasitol Res 119:4291-4295.

https://doi.org/10.1007/s00436-020-06928-5 PMID: 33057813

8. Dessi G, Tamponi C, Varcasia A, Sanna G, Pipia AP, Carta S, et al. (2020) *Cryptosporidium* infections in sheep farms from Italy. Parasitol Res 119:4211-4218. https://doi.org/10.1007/s00436-020-06947-2 PMID: 33140165

9. Thomson S, Hamilton CA, Hope JC, Katzer F, Mabbott NA, Morrison LJ, et al. (2017) Bovine cryptosporidiosis: impact, host-parasite interaction and control strategies. Vet Res 48:42. https://doi.org/10.1186/s13567-017-0447-0 PMID: 28800747

10. Caffarena RD, Meireles MV, Carrasco-Letelier L, Picasso-Risso C, Santana BN, Riet-Correa F, et al. (2020) Dairy calves in Uruguay are reservoirs of zoonotic subtypes of *Cryptosporidium parvum* and pose a potential risk of surface water contamination. Front Vet Sci 7:562. https://doi.org/10.3389/fvets.2020.00562 PMID: 32974408

11. Aberg M, Emanuelson U, Troell K, Bjorkman C (2020). A single-cohort study of *Cryptosporidium bovis* and *Cryptosporidium ryanae* in dairy cattle from birth to calving. Vet Parasitol Reg Stud Reports 20:100400. https://doi.org/doi: 10.1016/j.vprsr.2020.100400 PMID: 32448548

12. Yildirim A, Adanir R, Inci A, Yukari BA, Duzlu O, Onder Z, et al. (2020) Prevalence and genotyping of bovine *Cryptosporidium* species in the Mediterranean and Central Anatolia region of Turkey. Comp Immunol Microbiol Infect Dis 69:101425. https://doi.org/10.1016/j.cimid.2020.101425 PMID: 31978845

13. Liang N, Wu Y, Sun M, Chang Y, Lin X, Yu L, et al. (2019) Molecular epidemiology of *Cryptosporidium* spp. in dairy cattle in Guangdong Province, South China. Parasitology146:28-32. https://doi.org/10.1017/S0031182018001129 PMID: 29986775

14. Fayer R, Santin M, Xiao L (2005) *Cryptosporidium bovis n. sp.* (Apicomplexa: Cryptosporidiidae) in cattle (*Bos taurus*). J Parasitol 91:624-629. https://doi.org/10.1645/GE-3435 PMID: 16108557

15. Fayer R, Santin M, Trout JM (2008) *Cryptosporidium ryanae n. sp.* (Apicomplexa: Cryptosporidiidae) in cattle (*Bos taurus*). Vet Parasitol 156:191-198. https://doi.org/10.1016/j.vetpar.2008.05.024 PMID: 18583057

16. Peng MM, Wilson ML, Holland RE, Meshnick SR, Lal AA, Xiao L (2003) Genetic diversity of *Cryptosporidium* spp. in cattle in Michigan: implications for understanding the transmission dynamics. Parasitol Res 90:175-180. https://doi.org/10.1007/s00436-003-0834-5 PMID: 12783304

17. Khan A, Shaik JS, Grigg ME (2018) Genomics and molecular epidemiology of *Cryptosporidium* species. Acta Trop 184:1-14. https://doi.org/10.1016/j.actatropica.2017.10.023 PMID: 29111140

18. Lee YJ, Ryu JH, Shin SU, Choi KS (2019) Prevalence and molecular characterization of *Cryptosporidium* and *Giardia* in pre-weaned native calves in the Republic of Korea. Parasitol Res 118:3509-3517. https://doi.org/10.1007/s00436-019-06482-9 PMID: 31624910

19. Kabir MHB, Ceylan O, Ceylan C, Shehata AA, Bando H, Essa MI, et al. (2020) Molecular detection of genotypes and subtypes of *Cryptosporidium* infection in diarrheic calves, lambs, and goat kids from Turkey. Parasitol Int 79:102163. https://doi.org/10.1016/j.parint.2020.102163 PMID: 32589940

20. Silverlas C, Naslund K, Bjorkman C, Mattsson JG (2010) Molecular characterisation of *Cryptosporidium* isolates from Swedish dairy cattle in relation to age, diarrhoea and

region. Vet Parasitol 169:289-295. https://doi.org/10.1016/j.vetpar.2010.01.003 PMID: 20138705

21. Muhid A, Robertson I, Ng J, Ryan U (2011) Prevalence of and management factors contributing to *Cryptosporidium sp.* infection in pre-weaned and post-weaned calves in Johor, Malaysia. Exp Parasitol 127:534-538. https://doi.org/10.1016/j.exppara.2010.10.015 PMID: 21050848

22. Taylan-Ozkan A, Yasa-Duru S, Usluca S, Lysen C, Ye J, Roellig DM, et al. (2016) *Cryptosporidium* species and *Cryptosporidium parvum* subtypes in dairy calves and goat kids reared under traditional farming systems in Turkey. Exp Parasitol 170:16-20. https://doi.org/10.1016/j.exppara.2016.06.014 PMID: 27373430

23. Li N, Wang R, Cai M, Jiang W, Feng Y, Xiao L (2019) Outbreak of cryptosporidiosis due to *Cryptosporidium parvum* subtype IIdA19G1 in neonatal calves on a dairy farm in China. Int J Parasitol 49:569-577. https://doi.org/10.1016/j.ijpara.2019.02.006 PMID: 31071320

24. Lee SH, VanBik D, Kim HY, Lee YR, Kim JW, Chae M, et al. (2016) Multilocus typing of *Cryptosporidium* spp. in young calves with diarrhea in Korea. Vet Parasitol 229:81-89. https://doi.org/10.1016/j.vetpar.2016.09.019 PMID: 27809984

25. Kabir MHB, Itoh M, Shehata AA, Bando H, Fukuda Y, Murakoshi F, et al. (2020) Distribution of *Cryptosporidium* species isolated from diarrhoeic calves in Japan. Parasitol Int 78:102153. https://doi.org/10.1016/j.parint.2020.102153 PMID: 32504804

26. Cai M, Guo Y, Pan B, Li N, Wang X, Tang C, et al. (2017) Longitudinal monitoring of *Cryptosporidium* species in pre-weaned dairy calves on five farms in Shanghai, China.

Vet Parasitol 241:14-19. https://doi.org/10.1016/j.vetpar.2017.05.005 PMID: 28579024

27. Cheun HI, Choi TK, Chung GT, Cho SH, Lee YH, Kimata I, et al. (2007) Genotypic characterization of *cryptosporidium* oocysts isolated from healthy people in three different counties of Korea. J Vet Med Sci 69:1099-1101. https://doi.org/10.1292/jvms.69.1099 PMID: 17984603

28. Sulaiman IM, Hira PR, Zhou L, Al-Ali FM, Al-Shelahi FA, Shweiki HM, et al. (2005) Unique endemicity of cryptosporidiosis in children in Kuwait. J Clin Microbiol 43:2805-2809. https://doi.org/10.1128/JCM.43.6.2805-2809.2005 PMID: 15956401

29. Meganck V, Hoflack G, Piepers S, Opsomer G (2015) Evaluation of a protocol to reduce the incidence of neonatal calf diarrhoea on dairy herds. Prev Vet Med 118:64-70. https://doi.org/10.1016/j.prevetmed.2014.11.007 PMID: 25475689

30. Lee SH, Kim HY, Choi EW, Kim D (2019) Causative agents and epidemiology of diarrhea in Korean native calves. J Vet Sci 20:e64. https://doi.org/10.4142/jvs.2019.20.e64 PMID: 31775191

31. Avendano C, Ramo A, Vergara-Castiblanco C, Sanchez-Acedo C, Quilez J (2018) Genetic uniqueness of *Cryptosporidium parvum* from dairy calves in Colombia. Parasitol Res 117:1317-1323. https://doi.org/10.1007/s00436-018-5818-6 PMID: 29484550

32. Diaz P, Varcasia A, Pipia AP, Tamponi C, Sanna G, Prieto A, et al. (2018) Molecular characterisation and risk factor analysis of *Cryptosporidium* spp. in calves from Italy. Parasitol Res 117:3081-3090. https://doi.org/10.1007/s00436-018-6000-x PMID: 30008134

33. Holzhausen I, Lendner M, Gohring F, Steinhofel I, Daugschies A (2019) Distribution of *Cryptosporidium parvum gp60* subtypes in calf herds of Saxony, Germany. Parasitol Res 118:1549-1558. https://doi.org/10.1007/s00436-019-06266-1 PMID: 30790038

34. Lombardelli JA, Tomazic ML, Schnittger L, Tiranti KI (2019) Prevalence of *Cryptosporidium parvum* in dairy calves and *gp60* subtyping of diarrheic calves in Central Argentina. Parasitol Res 118:2079-2086. https://doi.org/10.1007/s00436-019-06366-y PMID: 31187226

35. Santoro A, Dorbek-Kolin E, Jeremejeva J, Tummeleht L, Orro T, Jokelainen P, et al. (2019) Molecular epidemiology of *Cryptosporidium* spp. in calves in Estonia: high prevalence of *Cryptosporidium parvum* shedding and 10 subtypes identified. Parasitology 146:261-267. https://doi.org/10.1017/S0031182018001348 PMID: 30086806

36. Wu Y, Zhang K, Zhang Y, Jing B, Chen Y, Xu C, et al. (2020) Genetic diversity of *Cryptosporidium parvum* in neonatal dairy calves in Xinjiang, China. Pathogens 9: 692. https://doi.org/10.3390/pathogens9090692 PMID: 32842484

37. Kaupke A, Rzezutka A (2015) Emergence of novel subtypes of *Cryptosporidium parvum* in calves in Poland. Parasitol Res 114:4709-4716. https://doi.org/10.1007/s00436-015-4719-1 PMID: 26358098

38. Qi M, Zhang K, Huang M, Wang S, Xu C, Wang T, et al. (2020) Longitudinal detection of *Cryptosporidium* spp. in 1-10-week-old dairy calves on a farm in Xinjiang, China. Parasitol Res 119:3839-3844. https://doi.org/10.1007/s00436-020-06904-z PMID: 32996049

39. Santin M, Trout JM, Xiao L, Zhou L, Greiner E, Fayer R (2004) Prevalence and age-

related variation of *Cryptosporidium* species and genotypes in dairy calves. Vet Parasitol 122:103-117. https://doi.org/10.1016/j.vetpar.2004.03.020 PMID: 15177715

40. Santin M (2013) Clinical and subclinical infections with *Cryptosporidium* in animals. N Z Vet J 61:1-10. https://doi.org/10.1080/00480169.2012.731681 PMID: 23134088

41. Feng Y, Xiao L (2017) Molecular epidemiology of cryptosporidiosis in China. Front Microbiol 8:1701. https://doi.org/10.3389/fmicb.2017.01701 PMID: 28932217

42. Tao W, Li Y, Yang H, Song M, Lu Y, Li W (2018) Widespread occurrence of zoonotic *Cryptosporidium* species and subtypes in dairy cattle from Northeast China: public health concerns. J Parasitol 104:10-17. https://doi.org/10.1645/17-140 PMID: 29088547

43. Wang R, Zhao G, Gong Y, Zhang L (2017) Advances and perspectives on the epidemiology of bovine *Cryptosporidium* in China in the past 30 years. Front Microbiol 8:1823. https://doi.org/10.3389/fmicb.2017.01823 PMID: 28979256

44. Bjorkman C, Lindstrom L, Oweson C, Ahola H, Troell K, Axen C (2015) *Cryptosporidium* infections in suckler herd beef calves. Parasitology142:1108-1114. https://doi.org/10.1017/S0031182015000426 PMID: 2589955

45. Feng Y, Ortega Y, He G, Das P, Xu M, Zhang X, et al. (2007) Wide geographic distribution of *Cryptosporidium bovis* and the deer-like genotype in bovines. Vet Parasitol 144:1-9. https://doi.org/10.1016/j.vetpar.2006.10.001 PMID: 17097231

46. Seppa-Lassila L, Orro T, Lassen B, Lasonen R, Autio T, Pelkonen S, et al. (2015) Intestinal pathogens, diarrhoea and acute phase proteins in naturally infected dairy calves. Comp Immunol Microbiol Infect Dis 41:10-16. https://doi.org/10.1016/j.cimid.2015.05.004 PMID: 26264522

47. Rieux A, Paraud C, Pors I, Chartier C (2013) Molecular characterization of *Cryposporidium* isolates from pre-weaned calves in Western France in relation to age. Vet Parasitol 197:7-12. https://doi.org/10.1016/j.vetpar.2013.05.001 PMID: 23735428

48. Xu Z, Li N, Guo Y, Feng Y, Xiao L (2020) Comparative genomic analysis of three intestinal species reveals reductions in secreted pathogenesis determinants in bovine-specific and non-pathogenic *Cryptosporidium* species. Microb Genom 6:e000379. https://doi.org/ 10.1099/mgen.0.000379 PMID: 32416746

49. Feng Y, Torres E, Li N, Wang L, Bowman D, Xiao L (2013) Population genetic characterisation of dominant *Cryptosporidium parvum* subtype IIaA15G2R1. Int J Parasitol 43:1141-1147. https://doi.org/10.1016/j.ijpara.2013.09.002 PMID: 24126186

50. Ichikawa-Seki M, Aita J, Masatani T, Suzuki M, Nitta Y, Tamayose G, et al. (2015) Molecular characterization of *Cryptosporidium parvum* from two different Japanese prefectures, Okinawa and Hokkaido. Parasitol Int 64:161-166. https://doi.org/ 10.1016/j.parint.2014.11.007 PMID: 25481361

51. Ramo A, Quilez J, Vergara-Castiblanco C, Monteagudo L, Del Cacho E, Clavel A (2015) Multilocus typing and population structure of *Cryptosporidium* from children in Zaragoza, Spain. Infect Genet Evol 31:190-197. https://doi.org/10.1016/j.meegid.2015.01.023 PMID: 25660036

52. Soba B, Logar J (2008) Genetic classification of *Cryptosporidium* isolates from humans and calves in Slovenia. Parasitology 135:1263-1270. https://doi.org/ 10.1017/S0031182008004800 PMID: 18664309

53. Valenzuela O, Gonzalez-Diaz M, Garibay-Escobar A, Burgara-Estrella A, Cano M, Durazo M, et al. (2014) Molecular characterization of *Cryptosporidium* spp. in

children from Mexico. PLoS One 9(4):e96128. https://doi.org/10.1371/journal.pone.0096128 PMID: 24755606

54. Trotz-Williams LA, Martin DS, Gatei W, Cama V, Peregrine AS, Martin SW, et al. (2006) Genotype and subtype analyses of *Cryptosporidium* isolates from dairy calves and humans in Ontario. Parasitol Res 99:346-352. https://doi.org/10.1007/s00436-006-0157-4 PMID: 16565813

55. Al Mawly J, Grinberg A, Velathanthiri N, French N (2015) Cross sectional study of prevalence, genetic diversity and zoonotic potential of *Cryptosporidium parvum* cycling in New Zealand dairy farms. Parasit Vectors 8:240. https://doi.org/10.1186/s13071-015-0855-9 PMID: 25896433

56. Grinberg A, Learmonth J, Kwan E, Pomroy W, Lopez Villalobos N, Gibson I, et al. (2008) Genetic diversity and zoonotic potential of *Cryptosporidium parvum* causing foal diarrhea. J Clin Microbiol 46:2396-2398. https://doi.org/10.1128/JCM.00936-08 PMID: 18508944

57. Inacio SV, Widmer G, de Brito RL, Zucatto AS, de Aquino MC, Oliveira BC, et al. (2017) First description of *Cryptosporidium hominis gp60* genotype IkA20G1 and *Cryptosporidium parvum gp60* genotypes IIaA18G3R1 and IIaA15G2R1 in foals in Brazil. Vet Parasitol 233:48-51. *gp60* https://doi.org/10.1016/j.vetpar.2016.11.021 PMID: 28043388

58. Ng JS, Pingault N, Gibbs R, Koehler A, Ryan U (2010) Molecular characterisation of *Cryptosporidium* outbreaks in Western and South Australia. Exp Parasitol 125:325-328. https://doi.org/10.1016/j.exppara.2010.02.012 PMID: 20219461

59. Waldron LS, Power ML (2011) Fluorescence analysis detects *gp60* subtype diversity

in *Cryptosporidium* infections. Infect Genet Evol 11:1388-1395. https://doi.org/10.1016/j.meegid.2011.05.008 PMID: 21609784

60. Zintl A, Proctor AF, Read C, Dewaal T, Shanaghy N, Fanning S, et al. (2009) The prevalence of *Cryptosporidium* species and subtypes in human faecal samples in Ireland. Epidemiol Infect 137:270-277. https://doi.org/10.1017/S0950268808000769 PMID: 18474128

61. Del Chierico F, Onori M, Di Bella S, Bordi E, Petrosillo N, Menichella D, et al. (2011) Cases of cryptosporidiosis co-infections in AIDS patients: a correlation between clinical presentation and *gp60* subgenotype lineages from aged formalin-fixed stool samples. Ann Trop Med Parasitol 105:339-349. https://doi.org/10.1179/1364859411Y.0000000025 PMID: 21929875

62. Glaberman S, Moore JE, Lowery CJ, Chalmers RM, Sulaiman I, Elwin K, et al. (2002) Three drinking-water-associated cryptosporidiosis outbreaks, Northern Ireland. Emerg Infect Dis 8:631-633. https://doi.org/10.3201/eid0806.010368 PMID: 12023922

63. Mammeri M, Chevillot A, Chenafi I, Thomas M, Julien C, Vallee I, et al. (2019) Molecular characterization of *Cryptosporidium* isolates from diarrheal dairy calves in France. Vet Parasitol Reg Stud Reports 18:100323. https://doi.org/10.1016/j.vprsr.2019.100323 PMID: 31796198

64. Thompson HP, Dooley JS, Kenny J, McCoy M, Lowery CJ, Moore JE, et al. (2007) Genotypes and subtypes of *Cryptosporidium* spp. in neonatal calves in Northern Ireland. Parasitol Res 100:619-624. https://doi.org/10.1007/s00436-006-0305-x PMID: 17031699

65. Waldron LS, Ferrari BC, Power ML (2009) Glycoprotein 60 diversity in *C. hominis*

and *C. parvum* causing human cryptosporidiosis in NSW, Australia. Exp Parasitol 122:124-127. https://doi.org/10.1016/j.exppara.2009.02.006 PMID: 19233175

66. Koinari M, Karl S, Ng-Hublin J, Lymbery AJ, Ryan UM (2013) Identification of novel and zoonotic *Cryptosporidium* species in fish from Papua New Guinea. Vet Parasitol 198:1-9. https://doi.org/10.1016/j.vetpar.2013.08.031 PMID: 24064001

67. Koinari M, Lymbery AJ, Ryan UM (2014) *Cryptosporidium* species in sheep and goats from Papua New Guinea. Exp Parasitol 141:134-137. https://doi.org/10.1016/j.exppara.2014.03.021 PMID: 24703974

68. Shrestha RD, Grinberg A, Dukkipati VS, Pleydell EJ, Prattley DJ, French NP (2014) Infections with multiple *Cryptosporidium* species and new genetic variants in young dairy calves on a farm located within a drinking water catchment area in New Zealand. Vet Parasitol 202:287-291. https://doi.org/10.1016/j.vetpar.2014.03.034 PMID: 24780161

69. Giangaspero A, Papini R, Marangi M, Koehler AV, Gasser RB (2014) *Cryptosporidium parvum* genotype IIa and *Giardia duodenalis* assemblage A in *Mytilus galloprovincialis* on sale at local food markets. Int J Food Microbiol 171:62-67. https://doi.org/10.1016/j.ijfoodmicro.2013.11.022 PMID: 24334090

70. Waldron LS, Dimeski B, Beggs PJ, Ferrari BC, Power ML (2011) Molecular epidemiology, spatiotemporal analysis, and ecology of sporadic human cryptosporidiosis in Australia. Appl Environ Microbiol 77:7757-7765. https://doi.org/10.1128/AEM.00615-11 PMID: 21908628

71. Deshpande AP, Jones BL, Connelly L, Pollock KG, Brownlie S, Alexander CL (2015) Molecular characterization of *Cryptosporidium parvum* isolates from human

cryptosporidiosis cases in Scotland. Parasitology 142:318-325. https://doi.org/10.1017/S0031182014001346 PMID: 25244937

72. Osman M, Benamrouz S, Guyot K, El Safadi D, Mallat H, Dabboussi F, et al. (2018) Molecular epidemiology of *Cryptosporidium* spp. in North Lebanon. J Infect Dev Ctries 12:34S. https://doi.org/10.3855/jidc.10014 PMID: 31805009

73. Mahfouz ME, Mira N, Amer S (2014) Prevalence and genotyping of *Cryptosporidium* spp. in farm animals in Egypt. J Vet Med Sci 76:1569-1575. https://doi.org/10.1292/jvms.14-0272 PMID: 25649937

74. Mi R, Wang X, Huang Y, Zhou P, Liu Y, Chen Y, et al. (2014) Prevalence and molecular characterization of *Cryptosporidium* in goats across four provincial level areas in China. PLoS One 9:e111164. https://doi.org/10.1371/journal.pone.0111164 PMID: 25343501

75. Naguib D, El-Gohary AH, Mohamed AA, Roellig DM, Arafat N, Xiao L (2018) Age patterns of *Cryptosporidium* species and *Giardia duodenalis* in dairy calves in Egypt. Parasitol Int 67:736-741. https://doi.org/10.1016/j.parint.2018.07.012 PMID: 30055334

76. Mercado R, Pena S, Ozaki LS, Fredes F, Godoy J (2015) Multiple *Cryptosporidium parvum* subtypes detected in a unique isolate of a Chilean neonatal calf with diarrhea. Parasitol Res 114:1985-19888. https://doi.org/10.1007/s00436-015-4364-8 PMID: 25673079

77. Al-Habsi K, Yang R, Williams A, Miller D, Ryan U, Jacobson C (2017) Zoonotic *Cryptosporidium* and *Giardia* shedding by captured rangeland goats. Vet Parasitol Reg Stud Reports 7:32-35. https://doi.org/10.1016/j.vprsr.2016.11.006 PMID: 31014653

78. Chalmers RM, Robinson G, Elwin K, Elson R (2019) Analysis of the *Cryptosporidium* spp. and *gp60* subtypes linked to human outbreaks of cryptosporidiosis in England and Wales, 2009 to 2017. Parasit Vectors 12:95. https://doi.org/10.1186/s13071-019-3354-6 PMID: 30867023

79. Connelly L, Craig BH, Jones B, Alexander CL (2013) Genetic diversity of *Cryptosporidium* spp. within a remote population of soay sheep on St. Kilda Islands, Scotland. Appl Environ Microbiol 79:2240-2246. https://doi.org/10.1128/AEM.02823-12 PMID: 23354707

80. Del Coco VF, Cordoba MA, Bilbao G, de Almeida Castro AP, Basualdo JA, Fayer R, et al. (2014) *Cryptosporidium parvum gp60* subtypes in dairy cattle from Buenos Aires, Argentina. Res Vet Sci 96:311-314. https://doi.org/10.1016/j.rvsc.2013.12.010 PMID: 24480390

81. Abeywardena H, Jex AR, Nolan MJ, Haydon SR, Stevens MA, McAnulty RW, et al. (2012) Genetic characterisation of *Cryptosporidium* and *Giardia* from dairy calves: discovery of species/genotypes consistent with those found in humans. Infect Genet Evol 12:1984-1993. https://doi.org/10.1016/j.meegid.2012.08.004 PMID: 22981927

82. Nolan MJ, Jex AR, Koehler AV, Haydon SR, Stevens MA, Gasser RB (2013) Molecular-based investigation of *Cryptosporidium* and *Giardia* from animals in water catchments in Southeastern Australia. Water Res 47:1726-1740. https://doi.org/10.1016/j.watres.2012.12.027 PMID: 23357792

83. O'Brien E, McInnes L, Ryan U (2008) *Cryptosporidium gp60* genotypes from humans and domesticated animals in Australia, North America and Europe. Exp Parasitol 118:118-121. https://doi.org/10.1016/j.exppara.2007.05.012 PMID: 17618622

84. Quilez J, Torres E, Chalmers RM, Robinson G, Del Cacho E, Sanchez-Acedo C (2008) *Cryptosporidium* species and subtype analysis from dairy calves in Spain. Parasitology 135:1613-1620. https://doi.org/10.1017/S0031182008005088 PMID: 18980704

85. Mirhashemi ME, Zintl A, Grant T, Lucy F, Mulcahy G, De Waal T (2016) Molecular epidemiology of *Cryptosporidium* species in livestock in Ireland. Vet Parasitol 216:18-22. https://doi.org/10.1016/j.vetpar.2015.12.002 PMID: 26801590

86. Santin M, Zarlenga DS (2009) A multiplex polymerase chain reaction assay to simultaneously distinguish *Cryptosporidium* species of veterinary and public health concern in cattle. Vet Parasitol 166:32-37. https://doi.org/10.1016/j.vetpar.2009.07.039 PMID: 19713046

Figure legend

Figure 1. Sequence comparisons between *C. bovis* and *C. ryanae* for the partial18S rRNA gene from Korean sequences obtained in this study and reference strains. Six nucleotide differences at 440, 460, 464–466, and 470 are shown. An asterisk indicates sequences obtained in this study.

Table 1. Prevalence and distribution of *Cryptosporidium* species according to age group in pre-weaned calves

Age (days)	Sample size	No. of positive (%)	95% CI	*Cryptosporidium* species (No.)		
				C. parvum	*C. ryanae*	*C. bovis*
1–10	271	53 (19.6%)	14.8–24.3	49	3	1
11–20	92	24 (26.1%)	17.1–35.1	23	1	0
21–30	39	3 (7.7%)	0.0–16.1	0	3	0
31–60	58	6 (10.3%)	2.5–18.2	0	5	1
Total	460	86 (18.7%)	15.1–22.3	72	12	2

Table 2. Distribution of *Cryptosporidium* species in pre-weaned Korean native calves according to age group

Age (days)	Frequency of *C. parvum* positivity (%)	χ^2 (*P*–value)	Frequency of *C. ryanae* positivity (%)	χ^2 (*P*–value)	Frequency of *C. bovis* positivity (%)	χ^2 (*P*–value)
1–10	49/271 (18.1%)	25.300 (0.000)	3/271 (1.1%)	16.020 (0.001)	1/271 (0.4%)	2.824 (0.419)
11–20	23/92 (25.0%)		1/92 (1.1%)		0	
21–30	0		3/39 (7.7%)		0	
31–60 (Ref.)	0		5/58 (8.6%)		1/58 (1.7%)	

Table 3. Distribution of *Cryptosporidium parvum* subtype according to age group

gp60 subtypes	Age groups (days)		No. of positive calves
	1–10	11–20	
IIaA14G1R1	1	0	1 (1.4%)
IIaA14G3R1	1	0	1 (1.4%)
IIaA15G1R1	1	0	1 (1.4%)
IIaA15G2R1	3	0	3 (4.2%)
IIaA16G4R1	1	1	2 (2.8%)
IIaA17G3R1	1	3	4 (5.6%)
IIaA17G4R1	1	1	2 (2.8%)
IIaA18G3R1	36	16	52 (72.2%)
IIaA19G1R1	1	0	1 (1.4%)
IIaA19G3R1	0	2	2 (2.8%)
IIaA19G4R1	3	0	3 (4.2%)
Total	46	26	72

Figure 1

Click here to access/download;Figure;Fig. 1.tif

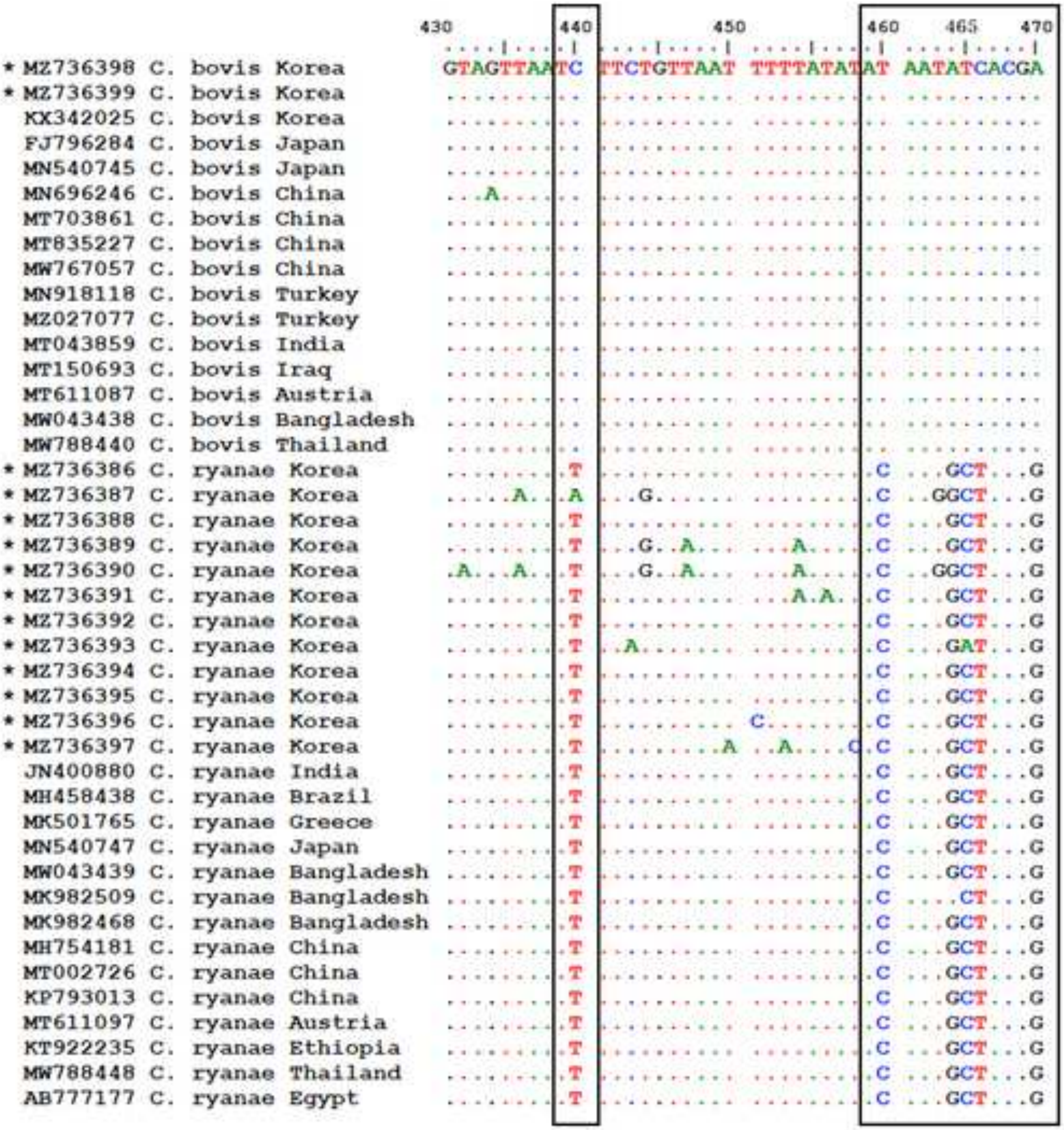

Supplement: Attachment — Submitted filename: PONE-D-21-26307_reviewer.pdf [file pone.0259824.s001.pdf]
