# Supplementary material for: Prevalence and distribution pattern of Cryptosporidium spp. among pre-weaned diarrheic calves in the Republic of Korea
Source: PLoS One. 2021 Nov 15;16(11):e0259824. doi: 10.1371/journal.pone.0259824 (PMC8592421; doi:10.1371/journal.pone.0259824)
Supplement: Attachment — Submitted filename: Response to Reviewers.docx [file pone.0259824.s002.docx]

**We thank the reviewers for their very careful reading and observations. These constructive comments are all valuable and very helpful for revising and improving our paper. We have detailed our response to points raised by the reviewer’s and modified our manuscript. Revised portions are marked in red in this manuscript**. **It would be greatly appreciated if our revised manuscript could be considered for publication in PLOS ONE.**

Reviewer #1: Dear Author,

I can say that I really like the subject of your article.
However, there is some confusion. What is the method by which you specify subtypes? According to which literature did you identify these subtypes?
I have not seen such a method in the literature number 24 that you use on this subject.
You should provide information about this in your article.

Kind regards

**Response: We are sorry for the confusion and agree with this comment. As the reviewer’s comment, we have provided them. Please see lines 131-133 and 466-468.**

Reviewer #2: The current MS presented Cryptospordiosis distribution among calves in ROK. The main issue of this work is the methods in which the authors described it in a briefly way. As PLOS ONE has broad base of readers, so, authors should be mentioned the methods used in something of details. Extraction of DNA from fecal samples needs specific precautions, authors need to mention how they manage these samples, add preservative or not, what about the inhibitors from feces? Also, I can't understand how the authors differentiate between C. ryanae and C. parvum? How did the authors decide that this is C. ryanae and other one is C. bovis then they did sequence and then differentiate between them?

**Response: We agree with this comment and understand the reviewer’s concern. Unfortunately, we did not examine oocysts from each feces, because we are not expert in this field. However, we used PCR method to detect *Cryptosporidium* species. All feces were stored at 4°C without additional treatment of preservation and were used for DNA extraction. We extracted directly DNA from feces using the QIAamp Fast DNA Stool Mini Kit (Qiagen) according to the manufacturer's instructions. We have provided the methods as the reviewer’s suggestion. Please see lines 121-126.**

**In addition, to detect *Cryptosporidium* species, we first used the 18S rRNA gene and sequenced all positive samples. By sequencing analysis, samples that yielded positive results for *Cryptosporidium* spp. were further screened to identify the species using species-specific primers. Positive samples for *C. parvum* was re-tested using the 60-kDa glycoprotein (*gp60*) gene to determine its subtype.** **To differentiate between *C. bovis* and *C. ryanae*, amplified sequences were compared and confirmed. According to the results of sequence, two species were different from each other. Please see lines 127-131, and 138-139.**

-The introduction needs a paragraph about importance and improvements that were done in the molecular identifications of Cryptosporidium.

**Response: In this study, we utilized the partial 18S rRNA gene, not SSU rRNA gene. In general, SSU rRNA gene is based on a nest PCR method. As you know, nested PCR has some problems such as contamination risks owing to multiple rounds of DNA amplification and concomitant DNA manipulation steps. To exclude these problems, we used conventional PCR method which have a short fragment. This method can easily be used to detect *Cryptosporidium* spp. We have provided them. Please see lines 87-91.**

- In the discussion the authors need to discusses why the age below 20 days calves are more susceptible to C. parvum?

**Response: We agree with this comment. At this point, we cannot make a conclusion about this, but it could be related to immune system of calves. In addition, it has been reported that *C. parvum* can be transmitted from cow to calf. Please see lines 219-227.**

Reviewer #3: Dear authors,
Thank you for this interesting work.
The manuscript is well written.
Here you are my comments/ remarks for your consideration.
Title
Line2
Should be changed: “Prevalence and distribution pattern of Cryptosporidium spp. among pre-weaned diarrheic calves in the Republic of Korea”

**Response: We have changed as the reviewer’s suggestion. Please see lines 2-3.**

Abstract
L24: Add Cryptosporidium spp. are protozoan parasites that belong to subphylum apicomplexa and cause diarrhea in humans and animals worldwide

**Response:** **We have modified as the reviewer’s suggestion. Please see lines 22-23.**

L42: Illustrate that family IIa belongs to C. parvum

**Response:** **We have modified as the reviewer’s suggestion. Please see lines 39-40.**

L45, 46: add the percentage of the rest subtypes that you found .

**Response:** **We have provided as the reviewer’s suggestion. Please see lines 43-45.**

Key words
L52 Add : spp. to Cryptosporidium,

**Response:** **We have modified as the reviewer’s suggestion. Please see line 51.**

Materials and Methods
L93-L131 The Materials and Methods should be written in more details.

**Response:** **We agree with this comment. As the reviewer’s comment, we have provided them. Please see lines 114-115, 117, 121-133, and 138-140.**

L110: Why did not you collect an equal number of samples in different age groups ?
Thus you can get more accurate prevalence and actual association between age and Cryptosporidium spp. distribution.

**Response: We understand the reviewer’s concern and agree with this comment. Our lab is focused on calf diarrhea, especially neonates. As you know, calf diarrhea occurs mainly less than 1 month old. Because most of the samples requested to be tested by our lab are under 30-day-old and feces were less in the age group over 30 days of age. For this reason, currently, it is difficult to compare the association between age group and distribution of *Cryptosporidium* spp. We are going to try to compare the prevalence and association between age and *Cryptosporidium* spp., if the number of samples is similar in all age groups. Please understand our situation. Thank you for your comment.**

Results and discussion
Results and discussion were written in a good details.

Reviewer #4: Dear Author
This is a generally well written manuscript with minor grammatical errors. Just a few comments or corrections:
1. Correlations are mentioned in the results (line 170) but not described in the statistical analysis.

**Response: We agree with this comment. We have provided. Please see lines 186-187 and Table 3.**

2. Consider moving the sentence in line 174 to the discussion as it is not a description of the results.

**Response: We agree with this comment. We have removed it and added in discussion. Please see lines 311-312.**

3. Line 216 statement on prevalence of C. ryanae in calves is ambiguous please revise.

**Response: We are sorry for the confusion and have revised. Please see lines 236-238.**

4. Line 296 change "sequencing analysis" to "sequence analyses".

**Response: We have changed as the reviewer’s suggestion. Please see lines 317-318.**

5. Line 297 add the word "respectively after the G at the end of the sentence. Line 313 add a recommendation.

**Response: We have modified as the** **reviewer’s suggestion and added some references. Please see line 319 and 335.**

Thank you and well done on this work.

Reviewer comment

1. Line 111. No microscopic examination was performed. How did you know for sure that the stools could be infective? Was it a coincidence?

**Response: We agree with this comment and understand the reviewer’s concern. We were not able to examine the oocytes because we are not expert in this field. So, we tested all fecal samples for detection of *Cryptosporidium* infection**.

2. Line 114. DNA was extracted from 200 mg. Specify what pretreatment was done to break up the oocyst wall.

**Response: We understand the reviewer’s concern. Because we did not test the oocysts and did not perform the step to break up the oocysts wall. To extract DNA, we directly used feces according to the manufacturer’s instructions. Please see lines 114-115, 117, and 121-126.**

3. Line 119. As far as I know, *C.bovis*/*ryanae* cannot be differentiated with this gene region. Clarification on the primers used should be clarified.

**Response: We agree with this comment. We have provided the reference of species-specific primers which we use. Please see line 129.**

4. Line 126. Since the base of sequences of C. bovis and C. ryanae are similar, all positive samples of the 18S rRNA were separated by comparing the sequences. Which method or according to which technique was this process done?

**Response:** We agree with this comment and understand the reviewer’s concern. **We first used the 18S rRNA gene to detect *Cryptosporidium* species and positive samples for *Cryptosporidium* species by sequencing analysis were screened to identify the four species using species-specific primers. Finally, positive samples for *C. bovis*/*C. ryanae* were differentiated by sequence analysis and species was confirmed based on the sequence analysis. According to our results, these two species had differences in nucleotide sequences.**

5. Line 142-143. If *C. andersoni* was not found, please specify.

**Response: We have provided. Please see lines 158-159.**
